# Target Nuclear Factor Erythroid 2-Related Factor 2 in Pulmonary Hypertension: Molecular Insight into Application

**DOI:** 10.1155/2022/7845503

**Published:** 2022-06-06

**Authors:** Yuhan Qin, Yong Qiao, Dong Wang, Linqing Li, Mingkang Li, Gaoliang Yan, Chengchun Tang

**Affiliations:** Department of Cardiology, Zhongda Hospital, School of Medicine, Southeast University, China

## Abstract

Nuclear factor erythroid 2-related factor 2 (Nrf2) is a key transcription factor involved in maintaining redox balance and activates the expression of downstream antioxidant enzymes. Nrf2 has received wide attention considering its crucial role in oxidative and electrophilic stress. Large amounts of studies have demonstrated the protective role of Nrf2 activation in various pulmonary hypertension (pH) models. Additionally, various kinds of natural phytochemicals acting as Nrf2 activators prevent the development of pH and provide a novel and promising therapeutic insight for the treatment of pH. In the current review, we give a brief introduction of Nrf2 and focus on the role and mechanism of Nrf2 in the pathophysiology of pH and then review the relevant research of Nrf2 agonists in pH in both experimental research and clinical trials.

## 1. Introduction

Pulmonary hypertension (pH) is a progressive life-threatening cardiopulmonary syndrome characterized by pulmonary vasoconstriction and pulmonary vascular remodeling (PVR) of pulmonary arterioles, leading to increased pulmonary artery pressure and pulmonary vascular resistance, culminating in right heart failure and even death [1]. Emerging evidence has shown the significant role of ROS in the pathogenesis of PH [2]. As a member of cap'n'collar basic-region leucine zipper transcription factor family, Nrf2 plays a critical role in sustaining cellular redox homeostasis through interconnecting effects [3]. Nrf2 regulates the transcription of large amounts of genes, including detoxifying enzymes, anti-inflammatory enzymes, stress response proteins, and metabolic enzymes [4]. Current researches have revealed decreased Nrf2 expression in pH; Nrf2 upregulation could remarkably ameliorate pulmonary vascular remodeling and right ventricular hypertrophy induced in a hypoxic pH experimental model [5]. The present review mainly focuses on the recent investigations on the therapeutic efficacy of Nrf2 agonists, particularly phytochemicals, in different types of pH and the underlying mechanisms.

## 2. Reactive Oxygen Species (ROS) in pH

Intima dysfunction, aberrant hyperplasia of vascular media, and dysregulated inflammation are the pathological basis of pH [6]. pH therapeutic approaches are mainly designed to target pulmonary vascular vasoconstriction, including proteinoids, endothelin receptor antagonists, and phosphodiesterase-5 inhibitors. These vasoactive agents attenuated the symptoms and delay the progression of pH. However, current approved drugs only confer modest benefits of mortality and life quality; pH remains an incurable disease [7]. The underlying mechanisms of pH are complicated; DNA damage response, endothelial-to-mesenchymal transition, metabolic reprogramming, inflammation, and epigenetic modification all participate in the pathogenesis and have become a focus in pH research field [8]. Various kinds of transcription factors, chemokines, cytokines, enzymes, and oxidative stress are involved in the pathogenesis of pH. Therefore, the underlying mechanism needs further elucidation, and a novel therapeutic target needs to be developed [9].

Oxidative stress response is activated to counter the harmful effects of redox imbalance. Altered redox status, enhanced oxidant stress, and ROS production were observed in the lung and right ventricular tissues of chronic hypoxia and monocrotaline (MCT) induced pH rat model as well as PAH patients, contributing to the development and progression of PH [10]. Emerging evidence has shown the generation of mitochondrial ROS; especially, superoxide mediates the pathology of pH, including acute pulmonary vasoconstriction, chronic pulmonary vascular remodeling, and right ventricular remodeling [11]. In particular, several NADPH oxidase (Nox) enzymes were identified as the main source of ROS [12]. Meanwhile, large amounts of antioxidant protective genes were expressed and provide benefit in the pH murine model, including detoxifying enzymes and antioxidant enzymes. Large amounts of studies showed ROS production is closely associated with various key characteristics of pH pathophysiology, including pulmonary vasoconstriction, pulmonary vascular remodeling, inflammation, and extracellular matrix remodeling [12]. Furthermore, increased ROS generation contributed to the hyperproliferation and apoptosis resistance and metabolic switch from oxidative phosphorylation to aerobic glycolysis in hypoxia-induced PASMCs [13]. ROS also stimulates inflammatory cascade and result to proliferation and apoptosis imbalance in PASMCs. Increased ROS production is also related to endothelial dysfunction in the PAH model induced by dasatinib [14]. Nox4-mediated ROS production in adventitial fibroblasts also contributed to hypertensive vascular remodeling and PAH development [15]. Current studies have been conducted to explore whether inhibition of oxidative stress through blockage of ROS-dependent signaling pathway or inhibition of ROS generation could prevent against the deleterious effects on pH and resultant right ventricular failure [16, 17].

## 3. Brief Introduction of Nuclear Factor Erythroid 2-Related Factor 2 (Nrf2)

ROS-producing enzymes and antioxidant enzymes are responsible for controlling intracellular ROS levels. In addition, both two kinds of enzymes are regulated by the pivotal transcription factor Nrf2.

Nrf2 is a member of cap'n'collar (CNC) basic-region leucine zipper (bZip) transcription factor family, which comprises nuclear factor erythroid-derived 2 (NFE2), Nrf1, Nrf2, and Nrf3 [18]. Nrf2 is encoded by NFE2L2 gene; it is a main sensor of oxidative stress and involved in sustaining cellular redox homeostasis, metabolism, and inflammation and is involved in the initiation of transcriptional regulation of downstream antioxidant enzymes. Nrf2 is increasingly recognized as a key transcription factor in protection against oxidative and electrophilic stress through modulating antioxidant protein expression [19]. Nrf2 induced the transcription of genes responsible for ROS detoxifying and damaged protein removal and contributed to cell survival [20]. Nrf2 regulates over 250 cytoprotective genes that present a regulatory enhancer sequence termed antioxidant response element (ARE). These genes construct a network participating in the regulation of antioxidant metabolism, inflammation, and the metabolism of carbohydrates, lipids, and iron [21]. Nrf2 is also essential for transcriptional induction of phase II enzymes, and Nrf2-knockout mice presented largely eliminated phase II enzymes in the liver and intestine [22]. Novel Nrf2 functions and targets have been identified, and Nrf2 relevant research is increasing. Pharmacological Nrf2 activation is a potential target for attenuation of ROS generation. Numerous Nrf2 activating compounds improved endothelial dysfunction and reduce oxidative stress. Previous findings indicated that targeting Nrf2-mediated ROS production might provide promising benefits in vascular oxidative stress [23].

Nrf2 has 7 functional domains termed Neh1-7, tangled in the regulation of its stability and transcriptional activity. NRF2 contains a bZip motif in Neh1, which is independent of heterodimerization with small musculoaponeurotic fibrosarcoma protein (sMAF) and activation of genes with ARE sequence. At the N-terminal domain, ETGE and DLG motifs in Neh2 are responsible for the combination between Nrf2 and Kelch-like epichlorohydrin-related protein 1 (Keap1), followed by Keap1-independent ubiquitination and degradation of Nrf2 in Neh 6 through binding to E3 ubiquitin ligase *β*-transducin repeat-containing protein (*β*TrCP) [24]. Neh3-5 domain activates transcriptional activity [25], while Neh7 mediates the combination with retinoic X receptor *α* and suppresses Nrf2 activity [26].

## 4. Keap1/Nrf2 Signaling Pathway

**Figure 1:**
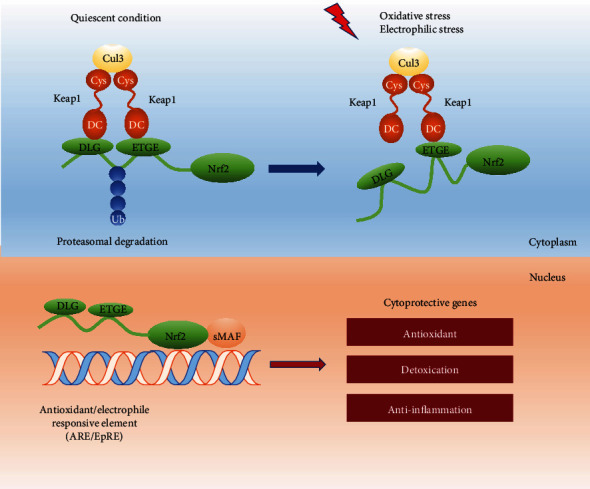
Keap1/Nrf2/ARE signaling pathway. Under unstressed condition, Nrf2 is degraded in Keap1-mediated ubiquitin proteasomal pathway. DLG and ETGE motifs in Nrf2 bind to DC domain of Keap1; the lysine residue between DLG and ETGE motif is the proteasomal degradation target, contributing to the low level of Nrf2 under quiescent conditions. Upon oxidative stress, the cysteine residues on Keap1 are inactivated by Nrf2 inducers, which hinder the degradation of Nrf2 and lead to increased Nrf2 expression. Accumulated Nrf2 translocates to the nucleus and heterodimerizes with sMAF proteins; downstream cytoprotective genes are activated through binding to the antioxidant response elements.

Cytoplasmic protein E3 ubiquitin ligase substrate adaptor Keap1 is the main upstream signaling pathway regulating Nrf2 expression. Keap1 belongs to the Kelch protein family and is a principal negative regulator of Nrf2 signaling pathway [27]. Nrf2-Keap1 system is essential for protection against oxidative and electrophilic stress via coordinated induction of a series of cytoprotective genes. Two Keap1 molecules form a homodimer and then bind to Nrf2 to form a trimer complex; this structure accelerates the proteasomal degradation of Nrf2 [28]. The two binding motifs ETGE and DLG in Neh2 domain of Nrf2 are responsible for recruiting Keap1 molecule and binding with DC domain of Keap1 [29]. Importantly, Keap1 acts as an essential bridge between Nrf2 and ubiquitination ligase Cullin-3 (Cul-3). Under quiescent conditions, Cul-3 is indispensable for the ubiquitination of lysine in Neh2 domain and the subsequent proteasomal degradation of Nrf2 by the 26S proteasome. Therefore, Nrf2 is sequestered in the cytoplasm with limited half-life at a very low level [30]. Inactivation of Keap1 strongly stimulates Nrf2 expression, which is often observed in chronic diseases, especially cancer. On exposure to oxidants or electrophiles, electrophiles and ROS react with distinct functioning cysteine sensors (C151, C226, C273, C288, and C613) of KEAP1; then sulfhydryl modifications of cysteine residues on Keap1 disrupted the proper conformation of Keap1-Nrf2-Cul3 complex [31]. Nrf2 activators disrupt the weakly interaction between Keap1 and DLG motif. Then, Nrf2 dissociates from Keap 1 and is prevented from ubiquitination degradation [32]. Nrf2 subsequently translocates to the nucleus, accumulating Nrf2 heterodimerizes with sMAFs, and promotes the transcriptional activity of ARE-containing cytoprotective genes [33, 34] (Figure 1). Recent studies have unveiled the Nrf2/Keap1 system is broadly involved in various biological processes, including cell proliferation and differentiation. Other cysteine-independent mechanisms have been found by interfering Nrf2/Keap1 complex through competitively binding to Keap1 or Nrf2 [35, 36]. *β*-TrCP-mediated nuclear Nrf2 degradation is another degradation pathway apart from Keap1/Nrf2 signaling. Mechanistically, the serine residue in Neh6 of nuclear Nrf2 is phosphorylated by glycogen synthase kinase 3, and then *β*-TrCP recognizes and captures phosphorylated Nrf2 and induces its proteasomal degradation [37].

## 5. Nrf2-Targeted Genes and Their Function

Nrf2-sMAF heterodimer induces numerous cytoprotective genes expression through binding to ARE or electrophile responsive element (EpRE) in the promoter region of Nrf2 target genes. ARE is essential for the recruitment of key transcription factors and characterized with the consensus sequence TGACNNNGC at 5′-neighboring regions [38]. Interestingly, the ARE of NFE2L2 promoter region contributes to the own activation of Nrf2, thereby amplifying the biological effects of Nrf2 in a positive feedback manner [39]. Nrf2-ARE signaling pathway plays an important role in protecting cells from physiological and pathophysiological injury through upregulation of a series of ARE dependent antioxidative genes and xenobiotic detoxifying enzyme expression [40].

Nrf2 regulates over 500 gene transcription encoding proteins acting as redox balancing factors, detoxifying and anti-inflammatory enzymes, stress response proteins, and metabolic enzymes and controls cellular homeostasis through interconnecting effects. The maintenance of intracellular redox homeostasis is mostly studied. Antioxidant and phase II detoxifying enzymes are key Nrf2 transcriptional products, including NAD (P)H:quinone dehydrogenase 1 (NQO1), heme oxygenase-1 (HO-1), glutathione S-transferase (GST), thioredoxin, thioredoxin reductase, glutamate-cysteine ligase catalytic subunit (GCLC), PRDX1 (Peroxiredoxin1), and other ROS scavengers. Chromatin immunoprecipitation sequencing (ChIP-Seq) analyses have discovered hundreds of novel cytoprotective target genes of Nrf2 [41].

HO-1 is a crucial stress-inducible antioxidant enzyme in the downstream of Nrf2 and confers tissue protection in multiple oxidative and inflammatory related diseases. HO-1 catalyzes the degradation of heme to bilirubin, carbon monoxide, and ferrous iron; thereby, these enzymatic end products contributed to the antioxidative effect of HO-1 [42]. Alam et al. found the transcription of HO-1 is significantly enhanced 25–30-fold in the presence of Nrf2 in fibroblasts [43]. Nrf2 is a regulator of both GCLC and its modifier subunit (GCLM), and Nrf2 promotes GSH synthesis under cysteine-replete conditions. The expression of GCLC and GCLM was associated with high levels of Nrf2 [44].

NQO1 is one of the main target genes of rfF2, and NRF2/NQO1 axis activates the antioxidant response and accelerates HO-1 expression. Upregulated NQO1 and HO-1 induced by Nrf2 activation showed a cellular protective effect [45].

## 6. Nrf2-Mediated Oxidative Stress and pH

pH is a progressive disease characterized by pulmonary vascular remodeling (PVR) and increased pulmonary vascular pressure. Vascular media thickening, stenosis of vascular lumen, and muscularization of pulmonary vessels contribute to the development of PVR, increased pulmonary artery pressure, leading to right heart failure [46]. Emerging evidence has suggested decreased Nrf2 contributed to the pathogenesis of PAH. It was demonstrated that expression of the Nrf2-regulated antioxidant enzymes was decreased in a patient with chronic obstructive pulmonary disease associated with pH. The pharmacologically or genetically induced Nrf2 activity clearly decreased right ventricular hypertrophy (RVH) and pulmonary vascular remodeling in the hypoxic pH model [47].

Increasing evidence supports the enhanced generation of pathological levels of ROS in the injured pulmonary vasculature in PAH. The aberrant behavior of PAECs, PASMCs, fibroblasts, and immune cells partially resulted from overproduction of ROS and reactive nitrogen species (RNS) via posttranslational modification, including binding to soluble guanylate cyclase, cysteine residues, oxidation of cysteine, and methionine residues [48]. Dysregulated oxidative signaling caused by an excess of ROS has been heavily implicated in the pathophysiology of PAH and participated in the metabolic switch from mitochondrial oxidative phosphorylation to aerobic glycolysis [13]. Uncontrolled ROS generation was determined to play a role in PVR and PASMCs proliferation [49]. Mitochondria and NADPH oxidases have been suggested as sources of ROS generation in HPH. Therefore, maintenance redox homeostasis might provide a therapeutic strategy for normal cell function and reverse PVR. Recent findings indicated the involvement of Nox4 in the generation of ROS triggered PVR induced by chronic hypoxia and right ventricular failure [50]. Another research showed Nox1 was responsible for increased intracellular superoxide production and PASMCs proliferation in the MCT-induced pH model. Therefore, different Nox isoforms were involved in the regulation of PASMCs proliferation and migration in different types of PH [49] [51].

Thioredoxin-1 (Trx-1) is one of the main systems that control the cellular redox environment and exerts a regulatory effect in cell survival and apoptosis [52]. Research showed that MCT promoted the decreased expression of Trx-1 and Nrf2. Moreover, Trx-1 is an antioxidant and regulated by Nrf2 [53]. Downregulated Nrf2, increased lipid peroxidation, and decreased antioxidant capacity were found in the lung tissue of the MCT-induced pH model. Intriguingly, restoration of Nrf2 expression improved the hemodynamic parameters and oxidative stress biomarkers [54]. Hood et al. explored the underlying mechanism of PASMC proliferation induced by serotonin and demonstrated decreased Nrf-2 and catalase activity in PASMCs. Dysregulated Nrf2 led to increased oxidative modification of proteins, redox-sensitive pathway activation, and abnormal mitogenic responses [55]. Nrf-2 is potentially a key molecule in preserving right ventricular function caused by pH. Oxidative damage contributed to right ventricular failure; upregulated Nrf2 partially reserved PAH-induced cardiomyocyte apoptosis and RV fibrosis, thereby limiting RV damage [56].

## 7. Epigenetic Regulation of Nrf2 Signaling

The development of the epigenetic research field has provided a novel dimension to the understanding of Nrf2 signaling pathways without alterations to the DNA sequence. Keap1 is the best regulator of Nrf2 via promoting its proteasomal degradation. In addition to the regulation of Nrf2 protein stability, we provide an update on various phytochemicals that regulate NRF2 via DNA methylation, histone modifications, and noncoding RNAs [57]. Targeting the epigenetic mechanisms of Nrf2 represents an attractive therapeutic strategy.

DNA methylation is a dynamical and reversible process regulated by DNA methyltransferases (DNMTs) and DNA demethylation enzymes. An increasing number of studies revealed that altered DNA methylation plays a central role in regulating Nrf2 expression and oxidative stress. Several CpG islands have been identified in the promoter of NFE2L2, and abnormal enhanced hypermethylation of these GPG sites of Nrf2 promoter are associated with reduced Nrf2 expression [58]. Recently, in preclinical and human clinical trials, numerous types of natural phytochemicals and herbs have been identified as Nrf2 epigenetic activator and exert chemoprevention effect and anticancer potential against tumors through reversing the hypermethylated status in CPG promoter region of Nrf2 [59], including sulforaphane (SFN), curcumin, luteolin, and *γ*-tocopherol [60–63].

Resveratrol significantly upregulated Nrf2 expression and its target genes NQO1 and HO-1 dose dependently. Additionally, the attenuated cellular and mitochondrial oxidative stress and improved vasodilation after resveratrol treatment were diminished in Nrf2^−/−^ mice [64]. Resveratrol also provides therapeutic benefits on nonalcoholic fatty liver disease (NAFLD) through epigenetic modification and the Nrf2 activation. Mechanistically, resveratrol reversed the methylation status of the Nrf2 promoter in NAFLD mouse model and in HepG2 cells treated by high glucose, demethylated Nrf2-promoted Nrf2 and Nrf2-controlled antioxidant gene transcription, and inhibited ROS generation [65].

Apigenin (API) is a kind of dietary chemopreventive phytochemical agent. Paredes-Gonzalez and his colleagues investigated the potential epigenetic effect of API and discovered that API could enhance the nuclear translocation of Nrf2 and restore the decreased expression of Nrf2 through reversing the hypermethylated Nrf2 promoter at 15 GpG sites, coupled with reduced activity of DNMT1, DNMT3a, and DNMT3b as well as histone deacetylases (HDACs) [66].

The medicinal herb Radix Angelicae Sinensis (RAS, also named Danggui) is widely used in Asia.

Su et al. reported that RAS and its bioactive component Z-ligustilide (Lig) promoted endogenous Nrf2 expression and downstream target genes, including HO-1, NQO1, and UGT1A1 in TRAMP mice. Bisulfite genomic sequencing and methylation DNA immunoprecipitation revealed that Lig and RAS treatment prevented DNA methylation in the first five CpGs of the Nrf2 promoter region and inhibited DNA methyltransferase activity in vitro. Collectively, these results suggest that Lig and RAS are able to demethylate the Nrf2 promoter CpGs, resulting in the restoration of Nrf2 and Nrf2 target genes [67].

Histone modification is another key epigenetic mechanism involved in Nrf2 mediated oxidative stress. The underlying mechanism of histone modification of Nrf2 has been widely elucidated in prostate cancer. Previous research reported Nrf2 is partly suppressed epigenetically by histone modifications in the prostate tumor of TRAMP mice. ChIP assays revealed increased binding trimethyl histone H3 (Lys9) protein to the CpG sites in TRAMP C1 cells. Moreover, Nrf2 levels could be reversed by HDAC inhibitors [68]. The anticancer effect of SFN and corosolic acid (CRA) was partially attributed to restored Nrf2 expression caused by altered histone modification. Specifically, SFN attenuated the protein expression of HDAC 1, 4, 5, and 7 while increasing the level of acetyl-Histone 3 (Ac-H3). ChIP assay revealed CRA upregulated acetylation of histone H3 lysine 27 (H3K27ac) and downregulated trimethylation of H3K27 in the Nrf2 promoter region, inducing the expression of impaired Nrf2 [69, 70]. Histone H3 lysine 27 (H3K27me3) trimethyltransferase EZH2 was associated with decreased Nrf2 level through upregulation of histone trimethylation at Nrf2 promoter [71].

The addition and removal of acetylation modification are crucial in the function of Nrf2. Acetylation of Nrf2 at Neh1 DNA-binding domain is necessary for Nrf2-dependent gene transcription [72]. HDAC2 deacetylated lysine residues of Nrf2 and thereby prevented Nrf2 protein from degradation [73]. Altered HDAC expression partially contributed to the restored Nrf2 level treated by API [66]. Su et al. indicated SFN exerts its cardioprotective effect through preventing Ang II-induced cardiac inflammation, oxidative damage, fibrosis, cardiac remodeling, and dysfunction, coupled with activation of Nrf2. They also revealed SFN promoted Ac-H3 accumulation in Nrf2 promoter region, accompanied by decreased HDAC activity and HDAC enzyme expression [74].

Various noncoding RNAs including microRNAs (miRNAs) and long noncoding RNAs (lncRNAs) involved in the regulation of Nrf2. Numerous miRNAs are widely recognized negatively regulated Nrf2 levels by targeting the 3′ untranslated region in podocytes, neuroblastoma cells, erythroid cells, and HepG2 cells, including miR-27a, miR-28, miR-34, miR-93, miR-128, miR-142-5p, miR-144, and miR-153 [75]. Increased miRNAs target Nrf2 and accelerate the degradation of Nrf2 mRNA, leading to an impaired antioxidative response [76]. Furthermore, miR-140-5p directly sponges Nrf2 and promotes myocardial oxidative damage in doxorubicin-induced cardiotoxicity [77]. Ashrafizadeh et al. reviewed over 20 miRNAs directly or indirectly involved in the regulation of Nrf2 pathway [78]. In terms of pH, miR23a was selected as the significantly expressed miRNA in idiopathic pulmonary artery hypertension patients and related to the pulmonary function of pH patients. miR23a inhibition resulted in an increase of Nrf2, suggesting miR23a might be an upstream regulator of Nrf2 [79]. lncRNA MIAT/miR-29a-5p axis might stimulate oxidative stress response in the HPH model through regulation of Nrf2 pathway [80]. Additionally, overexpression of small nucleolar RNA ACA11 was linked to Nrf2 nuclear import and reactive oxygen species generation.

RNA binding proteins mediated posttranscriptional modification is also involved in the regulation of Nrf2. HuR and AUF1 activated Nrf2 in different manner. HuR promotes Nrf2 maturation and nuclear export, while AUF1 enhances the stability of Nrf2 mRNA [81]. Moreover, transcript mutations of Nrf2 could also interfere Nrf2's binding to Keap1 and hinder its degradation [82].

## 8. Nrf2 Activators as a Novel Therapeutic Approach for pH

Oxidative stress and inflammation are the two key mechanisms underlying the pathogenesis of pH. Oxidative stress and ROS were found accumulated in the lung tissues of pH, which contributes to the pathogenesis and progression of cardiac and pulmonary changes in chronic hypoxia mediated PH [83]. Experimental evidence has illustrated that persistent inflammation, including numerous inflammatory cell infiltrations, proinflammatory cytokines, and chemokines, was all closely correlated with various types of PH [84]. Given the essential role of Nrf2 signaling pathway in protection against oxidative and inflammatory stress, researchers hypothesized that reinforcement of homeostasis through pharmacological activation and upregulation of Nrf2 might become an innovative approach for the treatment of pH. A previous report has demonstrated that Nrf2-deficient mice developed more severe RVH, while Keap1 knockdown mice presented improved PVR compared with WT mice after 3-week hypoxia exposure [85]. Of note, several types of phytochemicals have been identified as effective Nrf2 inducers and prevent pH development in vitro and in vivo, providing a novel insight into therapeutic targets for pH.

Resveratrol is a natural stilbenoid polyphenol widely present in many fruits and has antioxidant, anti-inflammatory, antitumor, and estrogenic activities [86]. Convincing supportive evidence has suggested the promising therapeutic prospect of resveratrol in pH. Resveratrol shows cardiovascular beneficial effects on pH by attenuating PASMC proliferation and ameliorating endothelial dysfunction through regulation of multiple signaling pathways, including suppression of SphK1/S1P-mediated NF-*κ*B activation and normalization of BMP/SMAD signaling pathways [87, 88]. Especially, the progression of hypertension in SHR rats was attenuated through restoration of Nrf2 expression and increased antioxidant potential after resveratrol administration [89]. However, it remains unknown whether resveratrol could ameliorate the progression of pH through targeting Nrf2-mediated oxidative stress.

SFN is an isothiocyanate and abundantly presented in various kinds of cruciferous vegetables. It possesses antioxidant, anti-inflammatory, and antitumor biological activities [90]. Studies showed sulforaphane acts as an effectively strong Nrf2 activator and motivates Nrf2-mediated anti-inflammatory pathways [91]. Mechanistically, SFN demethylation modification at the CpG islands of Nrf2 promoter region, leading to upregulation of Nrf2 gene expression via modulation of the expression of DNA methyltransferases, exhibits anticancer potential in colon cancer [92] and skin neoplasm [62]. Intriguingly, sulforaphane partially rescued SuHx-induced RV dysfunction and normalized increased right ventricular systolic pressure. Right ventricular hypertrophy, fibrosis, inflammation, and PVR were all ameliorated after sulforaphane treatment. Sulforaphane might have a direct effect on PVR and then reduce RV afterload, as well as directly improved RV function. Nrf2 and the downstream gene NQO1 were found increased in the right ventricle after administration of SFN [93]. Zhang and his colleagues further investigated whether the protective effect of SFN on pH is Nrf2 dependent. The findings revealed that Nrf2 knockout mice present RV diastolic dysfunction earlier than wild mice induced by SU5416 and 10% hypoxia for 4 weeks. SFN partially/completely reversed RV diastolic and systolic dysfunction, while the protective effect disappeared in Nrf2 knockout mice [94]. Therefore, Nrf2 exerted an essential role in SFN-medicated RV dysfunction and PVR, and Nrf2 activation might provide a novel therapeutic approach for pH treatment.

Dimethyl fumarate (DMF) is an FDA-approved first-in-class potential Nrf2 pathway activating agent exhibiting excellent anti-inflammation and antioxidative stress effect, and it has been explored in an experimental HPH model. Intriguingly, preclinical results demonstrated DMF provided hemodynamic benefit, ameliorated pulmonary vascular muscularization, and prevented the development of lung fibrosis in the HPH mouse model [95]. Excitingly, agonists of the Nrf2 signaling pathway, DMF, have entered the clinical study phase for the treatment of patients with pulmonary hypertension caused by systemic sclerosis (SSc) [96]. Regrettably, a double-blind, randomized, placebo-controlled trial demonstrated that SSc-PAH patients tolerated DMF poorly, and nonsignificantly reduced decline in 6-minute walking distance was found after 24 weeks of DMF treatment (-7.07% vs. -14.97%). Large sample clinical trials need to be conducted to further evaluate the tolerance and efficacy of DMF in PAH [97]. Muralidharan and his colleagues successfully designed advanced inhalable dry powders containing DMF for targeted delivery to the lungs using particle engineering design technology. Predictive lung deposition modeling showed the capability of these DMF particles to reach the lower airways to treat inflammation in pH and other pulmonary diseases [98].

Pachymic acid (PA) is a kind of traditional Chinese medicine and one of the main components of Poria cocos. The extensive pharmacological effect of PA has attracted the attention researchers, including anti-inflammatory, antioxidation, and insulin-like effects, and exhibits low toxicity [99]. Previous research reported PA inhibits the proliferation of gastric tumor cells [100]. Besides, PA attenuated sepsis-induced acute kidney injury via activation of Nrf2/HO-1 pathway. He and his colleges demonstrated that PA pretreatment could effectively ameliorate pulmonary vascular remodeling and dose dependently inhibit hypoxia-induced PASMC proliferation via activating Nrf2-Keap1-ARE signaling pathway. Both cytoplasmic and nuclear Nrf2 expression levels were significantly diminished in PASMCs induced by hypoxia, while hypoxia promoted Keap1 accumulation and PA reversed the dysregulated Nrf2 and Keap1 expression caused by hypoxia in PASMCs. PA treatment also upregulated the decreased Nrf2 downstream gene expression, including HO-1 and SOD-1, and reduced intracellular ROS generation [101]. The research findings demonstrated that PA might become a potential pH treatment approach.

Polyphenol has been demonstrated as a class of Nrf2 activator through binding with keap1 and inhibiting Keap1-mediated Nrf2 degradation [32]. Salvianolic acid A (SAA) is a kind of natural polyphenol antioxidant and has attracted increasing attention due to its oxidative and antifibrotic effect on various diseases [102]. Intriguingly, SAA administration ameliorated PVR, lung apoptosis, lung fibrosis, and myocardial hypertrophy in the MCT-induced PAH rat model [103]. Endothelial-to-mesenchymal transition (EndMT) has been demonstrated to be involved in PVR. Oxidative and inflammatory stress in PAECs partially contributed to development of EndMT [104]. Furthermore, SAA attenuated ROS production and protected PAECs against hypoxia-induced EndMT [105]. Another research suggested that SAA reduced oxidative stress-associated EndMT and improved pulmonary vascular function. Mechanistically, SAA acts as a direct radical scavenger and suppresses ROS generation via inhibiting the expression of ROS-producing enzyme Nox4 [106]. Additionally, SAA enhanced Nrf2 translocation and activation, subsequently leading to increased downstream antioxidative gene HO-1 expression [107].

Acosta and colleagues successfully produced 4 different inhalable powders of simvastatin (Nrf2 activator). The inhalable nanostructured microparticle dry powders of simvastatin were developed for targeted pulmonary delivery through advanced particle engineering design technology, and all powders successfully aerosolized with dry powder inhalers (DPI) human devices. They reported for the first time that inhaling simvastatin for the treatment of pH is safe and effective. The results showed that inhaled simvastatin as an aerosol could restore the dysregulated endothelial function and reduce pulmonary vascular resistance in pH rat model. Pulmonary artery pressure was significantly decreased from 25.2 mmHg to 21.6 mmHg after simvastatin inhalation [108].

Andrographolide (ANDRO) is a natural labdane diterpene lactone possessing anti-inflammatory, antitumor, and antiproliferation activity [109]. The antiproliferative activity of ANDRO prompted Nie to investigate the potential therapeutic effects of anti-inflammatory and antioxidant agent ANDRO on hypoxia-induced PVR. ANDRO ameliorated distal pulmonary arteries remodeling, right ventricular hypertrophy. In vitro, ANDRO decreased cell viability, proliferation, and migration and promoted cell apoptosis in PASMCs. Nrf2 was downregulated, whereas ROS-generating enzyme NOX was upregulated, contributing to rapid ROS production in PAH-PASMCs compared with healthy control. ANDRO treatment blocked ROS generation by suppressing NOX activation and augmented Nrf2 expression. NOX/Nrf2-mediated oxidative stress and inflammation might be involved in the underlying mechanism ANDRO reversing PVR [110].

Blueberry (BB) is a natural antioxidant agent and presents therapeutic effect in PAH. BB improved echocardiography and catheterization parameters in the MCT-induced PAH model. BB improved the blood flow across the tricuspid valve and decreased the PAP in the MCT-induced pH model [111]. BB attenuated ROS generation and lipid peroxidation and improved the redox state in right ventricle of pH rats [112]. Furthermore, BB restored the decreased Nrf2 expression in lung tissues of pH rats and improved pulmonary redox state [111]. The findings provide a basis for natural antioxidant interventions as a novel treatment approach in PAH.

2-Cyano-3,12-dioxooleana-1,9-dien-28-oic acid (CDDO) is a kind of derivatives of synthetic triterpenoid development for the treatment of inflammation and cancer. As the derivative of CDDO, Bardoxolone methyl (CDDO-Me) is more potent in the activation of Keap1/Nrf2/ARE pathway. The *α*,*β*-unsaturated carbonyl groups on CDDO-Me are responsible for the combination with cysteine residues of Keap1, leading to Nrf2 release from Keap1 and translocation to nucleus, then thereby triggering antioxidant and anti-inflammatory response [113]. Patients with advanced COPD had declined Nrf2 and Nrf2 genetic deletion in mice led to early-onset and severe emphysema. In addition, Nrf2 activator CDDO remarkably reduced oxidative stress in lung tissue and improved pH caused by cigarette smoke. Therefore, targeting Nrf2 might become a promising therapeutic approach for COPD and subsequent pH [114]. CDDO-Me has been applied in clinical trial of PAH due to its antioxidant, anti-inflammatory, antifibrosis, and antiproliferative properties [115]. A novel designed hybrid combining CDDO and nitric oxide could significantly attenuate PVR, right ventricular hypertrophy, and vascular muscularization in MCT-induced PH rats, suggesting it may be a promising agent for PAH intervention [116]. A phase II clinical trial for PAH patients due to interstitial lung disease without cardiac risk factors was conducted, and the interim data showed that CDDO-Me was well-tolerated and improved six-minute walk distance in PAH patients [117].

Ligustrazine is an active alkaloid obtained from Chinese herbs and widely used for occlusive cardiovascular and cerebral diseases and PAH. Ligustrazine is a vasodilator, and the underlying mechanisms include calcium antagonism, cAMP production, and No release. Recently, ligustrazine was identified as a Nrf2 activator and exerts antioxidant effects. Moreover, Muralidharan et al. reported for the first time that they developed inhaled TMP both liquid and dry powder inhalation aerosols. In vivo experiments showed safety and efficacy of inhaled TMP in reducing PAP in the MCT-induced pH model [118].

Mitochondrial oxidative phosphorylation is hindered in pH, and increased glucose levels are found in PAECs and PASMCs. Research reported metformin has positive effects in pH treatment. In addition, metformin is also a Nrf2 activator and has been shown to improve mitochondrial function in the pathology of various pulmonary diseases. Nanoparticle/microparticle metformin dry powder inhaler was developed, and it could be developed as a therapeutic treatment for pH [110]. Whether metformin could inhibit the progression of PH through Nrf2 activation remains unexplored and the underlying mechanisms needs to be further unraveled.

Oxymatrine is one of the central functional components of Chinese herb Kushen and has anti-inflammation and antioxidant properties. Specially, oxymatrine concentration in the lung and heart is markedly higher than other organs. Zhang et al. investigated whether oral oxymatrine treatment could bring benefit on the development of pH. The findings showed that administration of oxymatrine attenuated RVSP and PVR induced by MCT and hypoxia. In vitro experiment showed oxymatrine decreased the proliferation of PASMCs. Oxymatrine treatment reversed hypoxia induced Nrf2 downregulation, along with increased SOD1 and HO-1 expression, and downregulated hydroperoxide in PASMCs, indicating the antioxidant agent oxymatrine prevented the development of pH through activation of Nrf2 mediated anti-inflammatory and antioxidative response [119].  Table 1 presents a brief summary of these phytochemical Nrf2 activators in pulmonary hypertension.

Another nonphytochemical Nrf2 activator Oltipraz also displayed the therapeutic potential in pH. Oltipraz (50 mg/kg/d for 3 d) significantly led to the nuclear accumulation of Nrf2 and ameliorated right ventricular hypertrophy caused by hypoxia, while RVSP did not alter after oltipraz administration. Furthermore, the protective effect of oltipraz in pH was abolished in Nrf2^−/−^ mice, suggesting oltipraz exerted the therapeutic efficacy through Nrf2 activation [85].

Celastramycin was selected out from 5562 compounds using high-throughput screening system; it could inhibit PAH-PASMC proliferation effectively and safely. Mechanistically, celastramycin treatment increased Nrf2 expression, then subsequently reduced inflammation and cytosolic ROS levels, and improved mitochondrial energy metabolism in PAH-PASMCs. Celastramycin reduced the levels of cytosolic ROS through significant upregulation of ROS scavengers Nrf2, downregulation of NADPH oxidases, and slight increase in mitochondrial ROS in PAH-PASMCs [120].

## 9. Conclusion

Nrf2 is a master regulator of cellular response against oxidative stress and plays a significant role in protection against oxidative stress through regulation of the downstream antioxidant and anti-inflammatory genes expression. These gratifying research results on phytochemicals as activators of Nrf2 signaling are of great interest and might open avenues for exploring new therapeutic insights into the prevention and treatment of pH in the future. However, our understanding of the mechanisms underlying diet phytochemical needs further exploration due to the complicated molecule mechanism network. Further research is required to manipulate these mechanisms in a beneficial manner for disease interception.

## Figures and Tables

**Table 1:** Research of phytochemical Nrf2 activator in pulmonary hypertension.

Phytochemical	Dose	Administration route	Model	Mechanism	Benefit	Reference
Resveratrol	50 mg/l	Oral	Spontaneously hypertensive rats (SHR)	Restored Nrf2 and GST activity	Mitigated renal inflammation Reduced oxidative stress Attenuated the progression of hypertension in SHR.	[89]
SFN	0.5 mg/kg for 5 days	Subcutaneous injection	Su5416 and 10% hypoxia exposure for 4 weeks	Nrf2/NQO1 upregulation	Protected mice from developing and RV dysfunction	[93, 94]
DMF	90 mg/kg	Intraperitoneal	Chronic hypoxia and hypoxia/SU5416 mouse models	Inhibited proinflammatory NF*κ*B, STAT3 and cJUN signaling Promoted *β*TRCP-dependent proteasomal degradation of fibrogenic mediators *β*-catenin, TAZ and Sp1	Reversed hemodynamic changes Reduced inflammation, oxidative damage, and fibrosis Ameliorated vascular muscularization	[95]
DMF	Week 1: 120 mg qd Weeks 2-3: 120 mg bid Weeks 3-7: 120 mg qam 240 mg qpm Weeks 8-24: 240 mg twice	Oral	PAH patients associated with systemic sclerosis	—	Withdrawn after serious adverse event Nonsignificantly reduced decline in 6MWD (7.07% vs. -14.97%) from baseline to week 24 compared to placebo-treated subjects	[97]
PA	5 mg/kg per day	—	Hypoxia-induced PH model	Activated Nrf2-Keap1-ARE signaling pathway	Significantly reversed right ventricular hypertrophy and PVR Inhibited proliferation and promoted apoptosis in hypoxia-induced PASMCs	[101]
SAA	0.3, 1, and 3 mg/kg	Gavage	MCT-induced PAH model	Stimulated Nrf2 translocation and subsequent HO-1 upregulation	Attenuated EndMT, inhibited ROS formation. Improved vascular function and inhibited inflammation	[107]
Simvastatin	0.1% and 0.5% w/v	Inhalation	Shunt lamb model of PH	Nrf2 activation and RhoA/Rho kinase inhibition	Restored endothelial function and reduced PVR	[108]
ANDRO	1 mg/kg/day	Intraperitoneal	Chronic hypoxia- and Sugen/hypoxia-induced PH	Blocked ROS generation by suppressing NOX activation and augmenting Nrf2 expression.	Decreased PVR, mPAP, and right ventricular hypertrophy Reduced cell viability, proliferation and migration Increased PASMC apoptosis	[110]
BB	50, 100, and 200 mg/kg	Gavage	MCT-induced PH model	Restored Nrf2 expression in the lungs	Increased the E/A ratio across the tricuspid valve and tricuspid annular phase systolic excursion Decreased mPAP	[111]
CODO-NO	2.1 *μ*g/kg	Nose-only exposure system	MCT-induced pH model	Inhibited overproliferation of perivascular cells Diminished macrophage infiltration and oxidative stress by inactivation of NOX4.	Exhibited inhibition of pulmonary vasodilation and vascular remodeling Reduced cardiac hypertrophy and fibrosis	[116]
Ligustrazine	1% *w*/*v* in methanol	Inhalation	MCT induced PH model	Nrf2/ARE activation and a Rho/ROCK inhibition.	Significantly decreased RVSP	[118]
Oxymatrine	50 mg/kg/day	Oral	MCT and hypoxia induced PH model	Significantly upregulated Nrf2 and downstream SOD1, HO-1 expression Downregulated hydroperoxide levels	Attenuated RVSP and PVR	[119]
Oltipraz	50 mg/kg/d	Gavage	Hypoxia induced PH	Nrf2 activation	Ameliorated right ventricular hypertrophy	[85]
